# Supplementary material for: Identification of photoperiod-induced specific miRNAs in the adrenal glands of Sunite sheep (Ovis aries)
Source: Front Vet Sci. 2022 Jul 22;9:888207. doi: 10.3389/fvets.2022.888207 (PMC9354845; doi:10.3389/fvets.2022.888207)
Supplement: Supplementary file 1 [file Data_Sheet_1.ZIP › Supplementary file/miRDeep2 file.zip!/miRDeep2 file/Novel_Structure/LP42A2/Novel_658.pdf]

Provisional ID	:	NC_019484.2_38236
Score total	:	1.9
Score for star read(s)	:	-1.3
Score for read counts	:	0
Score for mfe	:	1.6
Score for randfold	:	1.6
Score for cons. seed	:	
Total read count	:	780
Mature read count	:	780
Loop read count	:	0
Star read count	:	0

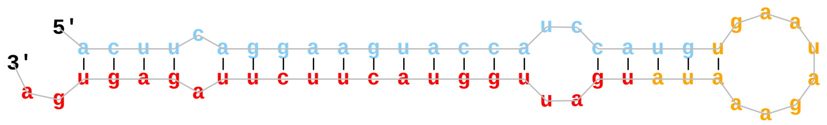

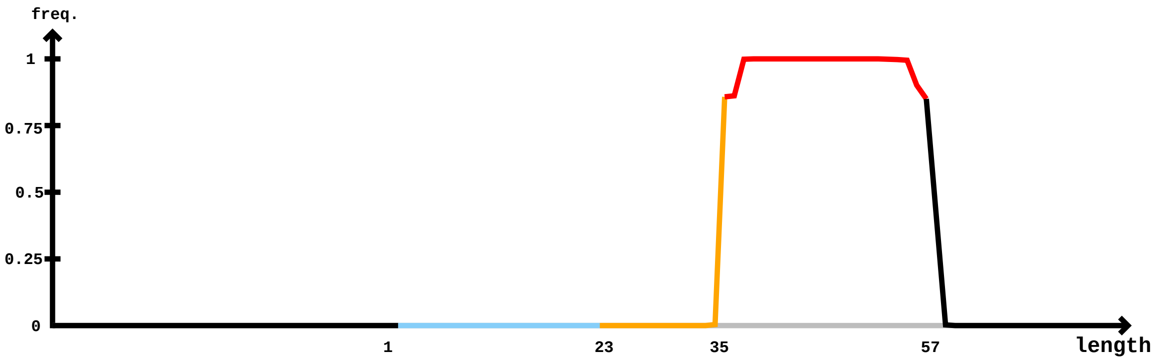

Star Mature

5'- ucaccacauuucacuaugccgcgugcaguauugcacuucaggaaguaccauccaugugaauagaaauaugauugguacuucuuagagugaaguaaugcauagcaaauaca -3'	exp		
................(((...(((((.((((.(((((.(((((((((((..(((((........)))))..))))))))))).))))).)))).))))).)))......	reads	mm	sample
....augauugguacuucuuagagu......................	2	0	seq
....augauugguacuucuuagagug.....................	1	0	seq
....ugauugguacuucuuag.........................	1	0	seq
....ugauugguacuucuuaga........................	1	0	seq
....ugauugguacuucuuagag.......................	2	0	seq
....ugauugguacuucuuagagu......................	71	0	seq
....ugauugguacuucuuagagug.....................	38	0	seq
....ugauugguacuucuuagaguga....................	503	0	seq
....ugauugguacuucuuagagugaa...................	49	0	seq
....ugauugguacuucuuagagugaag..................	1	0	seq
....gauugguacuucuuagaguga....................	3	0	seq
....auugguacuucuuagagug.....................	1	0	seq
....auugguacuucuuagaguga....................	78	0	seq
....auugguacuucuuagagugaa...................	27	0	seq
....auugguacuucuuagagugaag..................	1	0	seq
....uugguacuucuuagaguga....................	1	0	seq
